# An end-to-end hybrid algorithm for automated medication discrepancy detection

**DOI:** 10.1186/s12911-015-0160-8

**Published:** 2015-05-06

**Authors:** Qi Li, Stephen Andrew Spooner, Megan Kaiser, Nataline Lingren, Jessica Robbins, Todd Lingren, Huaxiu Tang, Imre Solti, Yizhao Ni

**Affiliations:** Department of Biomedical Informatics, Cincinnati Children’s Hospital Medical Center, 3333 Burnet Avenue, MLC 7024, Cincinnati, OH 45229-3039 USA; Chief Medical Information Officer, Cincinnati Children’s Hospital Medical Center, Cincinnati, OH USA; James M. Anderson Center for Health Systems Excellence, Cincinnati Children’s Hospital Medical Center, Cincinnati, OH USA

**Keywords:** Automated medication reconciliation, Medication discrepancy detection, Machine learning, Natural language processing

## Abstract

**Background:**

In this study we implemented and developed state-of-the-art machine learning (ML) and natural language processing (NLP) technologies and built a computerized algorithm for medication reconciliation. Our specific aims are: (1) to develop a computerized algorithm for medication discrepancy detection between patients’ discharge prescriptions (structured data) and medications documented in free-text clinical notes (unstructured data); and (2) to assess the performance of the algorithm on real-world medication reconciliation data.

**Methods:**

We collected clinical notes and discharge prescription lists for all 271 patients enrolled in the Complex Care Medical Home Program at Cincinnati Children’s Hospital Medical Center between 1/1/2010 and 12/31/2013. A double-annotated, gold-standard set of medication reconciliation data was created for this collection. We then developed a hybrid algorithm consisting of three processes: (1) a ML algorithm to identify medication entities from clinical notes, (2) a rule-based method to link medication names with their attributes, and (3) a NLP-based, hybrid approach to match medications with structured prescriptions in order to detect medication discrepancies. The performance was validated on the gold-standard medication reconciliation data, where precision (P), recall (R), F-value (F) and workload were assessed.

**Results:**

The hybrid algorithm achieved 95.0%/91.6%/93.3% of P/R/F on medication entity detection and 98.7%/99.4%/99.1% of P/R/F on attribute linkage. The medication matching achieved 92.4%/90.7%/91.5% (P/R/F) on identifying matched medications in the gold-standard and 88.6%/82.5%/85.5% (P/R/F) on discrepant medications. By combining all processes, the algorithm achieved 92.4%/90.7%/91.5% (P/R/F) and 71.5%/65.2%/68.2% (P/R/F) on identifying the matched and the discrepant medications, respectively. The error analysis on algorithm outputs identified challenges to be addressed in order to improve medication discrepancy detection.

**Conclusion:**

By leveraging ML and NLP technologies, an end-to-end, computerized algorithm achieves promising outcome in reconciling medications between clinical notes and discharge prescriptions.

**Electronic supplementary material:**

The online version of this article (doi:10.1186/s12911-015-0160-8) contains supplementary material, which is available to authorized users.

## Background

Several studies have reported the prevalence of the medication discrepancy problem in adult patients [1-3]. According to the most conservative estimate in the literature, about half of the adult and geriatric patients in primary care had at least one medication discrepancy [1,2]. The studies investigating the harm associated with medication discrepancies indicated that 30-90% of unintentional discrepancies upon hospital discharge had the potential to cause a significant clinical impact [1,3]. To improve medication accuracies, medication reconciliation, the process of comparing a patient’s medication orders to all medications the patient has been taking, is frequently utilized to detect medication discrepancies and then communicate the newly reconciled list to the patient and the clinical care providers [4,5]. In recent years, medication reconciliation has become common practice to prevent medication-related errors and is now an expected section of accreditation processes for medical institutions [1,6-21].

Despite its wide acceptance, medication reconciliation is inadequately performed in current clinical practice. Sustaining effective and accurate reconciliation remains challenging [22-27]. Literature studies identified various factors contributing to the inadequacy of medication reconciliation, among which two key findings are complexity of the reconciliation process and lack of time in a busy clinical practice setting [22,26,27]. In addition, many respondents noted that physicians frequently used free-text medication lists in clinical notes instead of using computerized provider order entry systems [21,28,29]. The free-text medication data is inaccessible to computerized reconciliation applications that rely on structured medication information, which further increases the medication reconciliation burden. As such, accurate and timely reconciliation during care transitions poses significant challenges to clinical care providers, and it has received the attention of both the World Health Organization and the Institute for Healthcare Improvement [30,31].

Initial efforts have been made to improve the efficacy of the medication reconciliation process, but most of them rely on manual vigilance and, subsequently, are prone to clinician fatigue and human errors [9,11,13,14]. Only a handful of studies have investigated automated or semi-automated approaches: Hassan et al. attempted to identify missing medications between patients’ medication lists using a collaborative filtering method and Silva et al. proposed a natural language processing- (NLP) based approach to reconcile medications manually identified from clinical notes to structured prescription lists [17,18]. A similar study was presented by Schnipper et al. on reconciling a patient’s preadmission prescription list with the discharge medication regimen in the Electronic Health Record (EHR) using a commercialized clinical decision support tool [19]. However, previous studies only reconciled medications between structured prescription lists; the challenges of reconciliation on free-text clinical notes remained unsolved. The recall in identifying matched and discrepant medications plateaued at less than 50% on the synthetic data and degraded when the approaches were evaluated in real-world scenarios (e.g. 23.4% recall on the matched medications reported in the work of Schnipper et al.) [17,19]. To reconcile medications documented in free-text clinical narratives, Cimino et al. used an external NLP system to parse clinical notes and identify medication terms, which were then matched to medication categories using a hard-coded medical entities dictionary [20]. However, the study was evaluated on only 17 patient records. Further development and evaluation of automated medication reconciliation is therefore required.

To address these barriers and fill the gap in knowledge, we developed and implemented state-of-the-art machine learning (ML) and NLP technologies and built a computerized algorithm for medication reconciliation. Our specific aims are: (1) to develop a hybrid automated algorithm for discrepancy detection between patients’ discharge prescriptions (structured data) and medications documented in free-text clinical notes (unstructured data); and (2) to assess the performance of the algorithm on gold-standard-based real-world medication reconciliation data. The overall objective is to develop an end-to-end computerized algorithm to identify potential medication discrepancies to reduce the pool of medications for manual reconciliation. Leveraging a double-annotated, physician-validated gold-standard set of medication reconciliation data, we demonstrated that an EHR-based computerized algorithm could improve medication discrepancy identification and substantially reduce the effort of manual reconciliation.

## Methods

In this study we focused on pediatric in patients enrolled in the Complex Care Medical Home Program (CCMHP) at Cincinnati Children’s Hospital Medical Center (CCHMC) between 1/1/2010 and 12/31/2013. Approval of ethics for this study was given by the CCHMC institutional review board (study ID: 2013–4241) and a waiver of consent was authorized.

### Data sources

**Figure 1:**
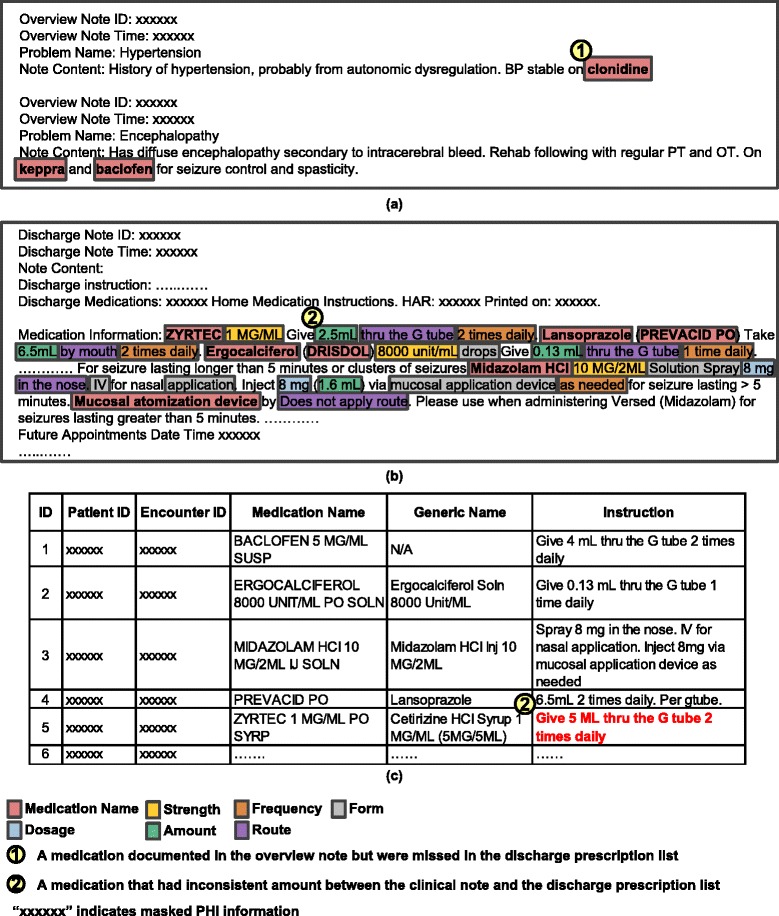
The example overview note **(a)**, discharge summary **(b)** and discharge prescription list **(c)** for an encounter. The medication information identified by the annotators is highlighted in clinical notes.

CCMHP serves a small population of patients with multiple chronic illnesses and dependence on medical technologies such as feeding tubes or artificial airways. The target population is an ideal group for medication reconciliation study because the patients usually have long medication lists, multiple care providers, and frequent transition between the hospital, clinic, and home care settings. Based on the pre-study communication with the physicians, we focused on patient discharges where two types of clinical notes (unstructured data) could be compared to the discharge prescription list (structured data). Our motivation arises from the fact that the medications documented in the free-text notes might be missed during the order-entry process. As such, the computerized reconciliation between the clinical notes and the prescription list could alert prescribers to medication discrepancies that would not otherwise be discovered. The clinical notes being reconciled included: (1) problem overview notes, which, by common practice in the CCMHP, described each patient’s problem and the corresponding plan of care including medications, and (2) discharge summaries, which described the post-discharge therapy including medications. Figure 1 shows an overview note, a discharge summary, and a prescription list example for a patient visit (encounter). Medications in clinical notes were mentioned in free text (Figure 1a and b). The information of discharge prescriptions was stored in multiple data fields in the EHR database (Figure 1c). We used SQL queries to extract all fields associated with discharge prescriptions, including patient ID and encounter ID in which a prescription was ordered, prescription name and generic name, instruction, route, frequency, strength, form, ordering date, and start and end dates. For each encounter, we extracted the overview notes associated with a patient’s problems that remained active at the time of discharge, suggesting that the patient should still take the medications for these problems. We also extracted all discharge summaries and the corresponding discharge prescription lists for the encounters. In total, we collected 4025 overview notes, 1717 discharge summaries and 975 prescription lists for all 975 inpatient encounters (271 complex care patients) during the study period. We manually reconciled all 4025 overview notes against the 975 prescription lists. Because discharge summaries are much longer and a comprehensive reconciliation of them was not feasible, we randomly sampled 300 (17.5%) discharge summaries and reconciled them against their corresponding prescription lists.

### Gold-standard medication reconciliation data

Medication matches and discrepancies between the clinical notes and the prescription lists were double annotated by two annotators using the Knowtator plug-in for Protégé [32,33]. Both annotators are English speakers (one clinical research nurse and one with an associate of applied science degree in health information science), with at least one year of clinical text annotation experience. Following the guidelines developed by the physicians, they first identified medication entities (e.g. medication name) from clinical notes and linked the attributes (amount, dosage, duration, form, frequency, route, and strength, highlighted in Figure 1a and b) to the corresponding medication names. Then they matched the identified medications with the prescriptions in the discharge list: (1) if the medication entities were identical between the notes and the prescriptions, they labeled them as “matched”; (2) if a medication in the notes was missed in the prescription list (e.g. Bullet one in Figure 1) or its medication attributes were not consistent (e.g. Bullet two), they labeled it as “discrepant”. Since a discharge prescription list also contained medications prescribed by other health care providers (documented upon hospital admission) which were not mentioned in the collected clinical notes, we did not reconcile medications in the reverse direction (matching discharge prescriptions with medications in the clinical notes) to avoid false positives of discrepant medications.

After the annotation process, differences between the annotators’ decisions were resolved under the supervision of an annotation manager (bachelor’s degree with more than four years of experience in clinical text annotation) and the inter-annotator agreement (IAA) was calculated using F-value to measure the agreement [34]. The consensus of the medication entities identified in the clinical notes, the associations between the attributes and the medication names, and the medication matches and discrepancies labeled by the annotators were then used as the gold-standard data to train and evaluate the automated algorithm.

### Automated medication discrepancy detection

**Figure 2:**
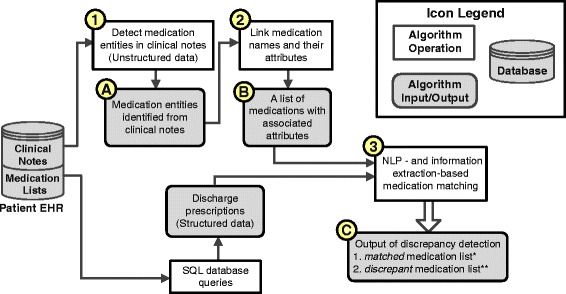
The architecture of the proposed automated medication discrepancy detection algorithm. Bullet 1–3 represent the three algorithm processes. Bullet **A-C** represent the outputs of the processes that were evaluated in the study. *“Matched medication list” includes the medication entities mentioned in the clinical notes and that had matches in the prescription list. **“Discrepant medication list” includes the medication entities mentioned in the clinical notes but were missed in the prescription list, or had medication attributes that were not consistent between the notes and the list.

The proposed medication discrepancy detection algorithm is diagramed in Figure 2. Given the clinical notes and the prescription list for an encounter, the algorithm first identified all medications and attributes from the clinical notes using ML techniques (Process 1 in Figure 2). It then linked the attributes to the corresponding medication names on the basis of a rule-based method (Process 2). Finally, a hybrid algorithm compared the identified medication entities with the prescriptions in the prescription list and returned a list of medications/attributes with associated “matched” or “discrepant” labels (Process 3).

#### Medication entity detection and attribute linkage

Medication entity detection, including medication name and attribute detection, was designed in-house based on the MALLET conditional random field (CRF) package [35,36]. Details of the medication entity detection process can be found in our earlier publications [37-39]. The process first tokenized and parsed the clinical notes with an in-house tokenizer and part-of-speech tagger. For each word, the token-level properties (e.g. capitalization and punctuation), the context (e.g. tokens before and after the studied token) and the part-of-speech tags were then used as text features in the CRF model. The term-level medication information, including the Concept Unique Identifiers from the Universal Medical Language System, Systematized Nomenclature of Medicine -- Clinical Terms codes, and the clinical drug codes of RxNorm, were also identified from the text using the clinical Text Analysis and Knowledge Extraction System and stored as medical term features in the CRF model [40]. Finally, the CRF model was trained on a set of clinical notes with gold-standard annotations (Figure 1) before executing entity detection on the held out test set (see “Experiments” section for the detailed setup of the experiments).

Attribute linkage applied a rule-based method to associate the identified attributes to the medication names [37]. The pseudo-code of the algorithm is presented in Table A.1 (Additional file 1). In summary, each identified attribute was linked to the closest medication name in absolute character distance, whether the medication preceded or followed the attribute.

#### Medication matching

**Figure 3:**
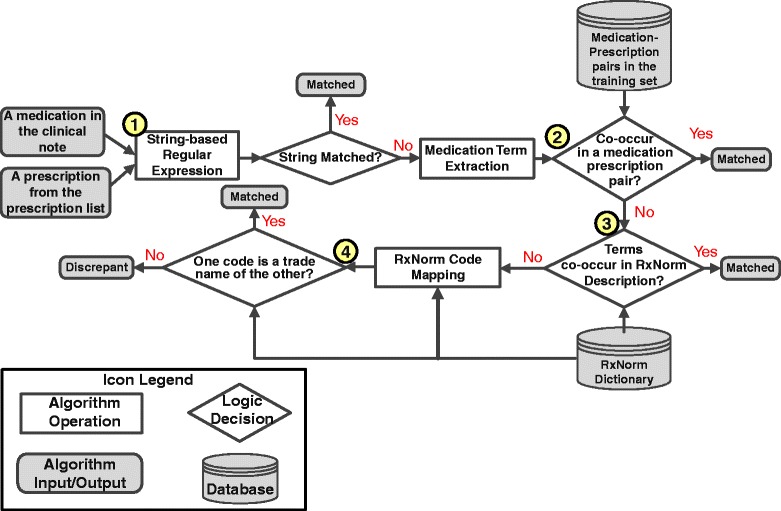
The diagram of the medication matching process.

Building on our previous experience in clinical trial-patient matching, the medication matching customized the NLP-based method in the literature and extended it with information extraction techniques [18,41]. The process reconciled each medication-prescription pairs with four steps, which are diagramed in Figure 3.

The first step (Bullet 1 in Figure 3) was a string-based regular expression matching. If the medication in the clinical notes was identical to, or a substring of, a prescription in the prescription list (or vice versa), the algorithm returned a “matched” label. If no match was found, the same step was also applied to compare the medication with the generic name of the prescription. If the exact match was not found, the mention of the medications (e.g. “ipratropium” and “albuterol” in “ipratropium 500 mg/2.5 ml – albuterol 2.5 mg/0.5 ml nebulizer solution”) was extracted from the medication identified in the notes and the prescription in the list. If these terms co-occurred in a medication-prescription pair previously observed in the training set, the algorithm returned “matched.” This second step utilizes the medication-prescription pairs in the training set for approximate term matching, which enables the algorithm to learn from previously collected information in practice. Step three was similar to step two but used the RxNorm dictionary to find medication synonyms [42]. If the medication terms co-occurred in the same RxNorm description (e.g. “diastat” and “diazepam” co-occurred in the description of RxNorm code 2052646 “diazepam rectal gel [diastat]”), implying that they were probable synonyms, the algorithm returned “matched.” The algorithm also utilized the RxNorm dictionary to match trade names of the medications (Step 4). In this step, both the medication and the prescription were mapped to the RxNorm codes using information extraction techniques. If one code was a trade name of the other, the algorithm returned “matched.” If no match was found from the above steps, the algorithm returned “discrepant.”

For each medication identified in the clinical notes, the matching process compared it with all prescriptions in a corresponding prescription list. If a matched prescription was found, the algorithm further compared the associated attributes with the prescription attributes using string-based regular expressions and output “matched” or “discrepant” label for each attribute.

### Experiments

#### Evaluation metrics

We used three standard NLP metrics to measure performance: precision = True Positive/(True Positive + False Positive), recall = True Positive/(True Positive + False Negative), and F-value = (2*P*R)/(P + R).

The experiments were conducted in a ten-fold cross validation setting, where the gold-standard data was divided at the encounter level and randomly split into ten rotating subsets – nine for training and one for testing at each run.

#### Performance tests of medication discrepancy detection

We evaluated the performances of the three processes individually and in combination to assess the error propagation through the algorithm pipeline. The outputs of the three processes are depicted in Figure 2 (Bullet A-C).

We first evaluated the performances individually. The CRF-based entity detection algorithm was trained and tested in the ten-fold cross validation setting. To ensure the unbiased assessment of the algorithm, in each fold we performed a stratified random sampling based on numbers of clinical notes for each patient to split the data into training and test sets. To assess the attribute linkage process, the gold-standard medication and attribute annotations were fed into the algorithm and the performance was evaluated against the gold-standard medication-attribute associations. The same setting was applied when assessing the medication matching process, where the performances were presented for gold-standard matched and discrepant medications respectively. A true positive of a matched medication was determined if and only if the algorithm returned a “matched” label and the medication was matched to the same prescription as identified by the annotators. Following the literature, the evaluations on the first two processes were assessed at the token level, while the evaluation on medication matching was assessed at the span level to ensure that each medication and its attributes were evaluated only once [37,39].

We then evaluated the performances that cumulatively integrated the processes. The output of the medication entity detector (Bullet A in Figure 2) was the input of the attribute linkage algorithm instead of the gold-standard annotations. Similarly, the medication names and the associated attributes used in medication matching also came from the predictions of the first two processes (Bullet B in Figure 2). Since the objective of this study was to develop a high-recall algorithm for detecting discrepant medications, we also analyzed the recall propagation on discrepant medications to assess the influence of each algorithm component on discrepant medication detection.

In both experiments no manual customizations were made to the automated algorithm to avoid over-fitting the current data sets. The entity detection and the medication matching algorithms were always trained on the data that was never part of the test set in each run of the experiments.

## Results

### Descriptive statistics of gold-standard data

Table 1 shows the descriptive statistics of the annotated corpus, including the number of patients, encounters, clinical notes and the numbers of matched and discrepant entities for each entity category. The annotators identified 29,596 entities from the clinical notes, where the discharge summaries had higher entity density than the overview notes (53 versus three entities per note). All medication attributes had similar frequencies except dosage and duration. The corpus had 7.5% of discrepant medications and attributes, scattered across 164 patients (60.5%) and 423 encounters (43.4%).

**Table 1:** **Descriptive statistics of the annotated clinical notes**

**Statistics**	**Overview notes**			**Discharge summaries**			**Overall**		
Encounters	975			168			975		
Patients	271			112			271		
Notes	4025			300			4325		
**Category**	**Discr**	**Match**	**All**	**Discr**	**Match**	**All**	**Discr**	**Match**	**All**
Medication name	1116	3873	4989	24	2935	2959	1140	6808	7968
Amount	169	1270	1439	47	1937	1984	216	3207	3423
Dosage	106	857	963	26	711	737	132	1568	1700
Duration	4	16	20	0	70	70	4	86	90
Form	36	1246	1282	6	2434	2440	42	3680	3722
Frequency	410	2742	3152	38	2635	2673	448	5377	5825
Route	48	443	491	42	2836	2878	90	3279	3369
Strength	142	1281	1423	4	2092	2096	146	3373	3519
Overall	2031	11728	13759	187	15650	15837	2218	27378	29596

“Discr” column shows the number of discrepant entities and “Match” the number of matched entities.

**Figure 4:**
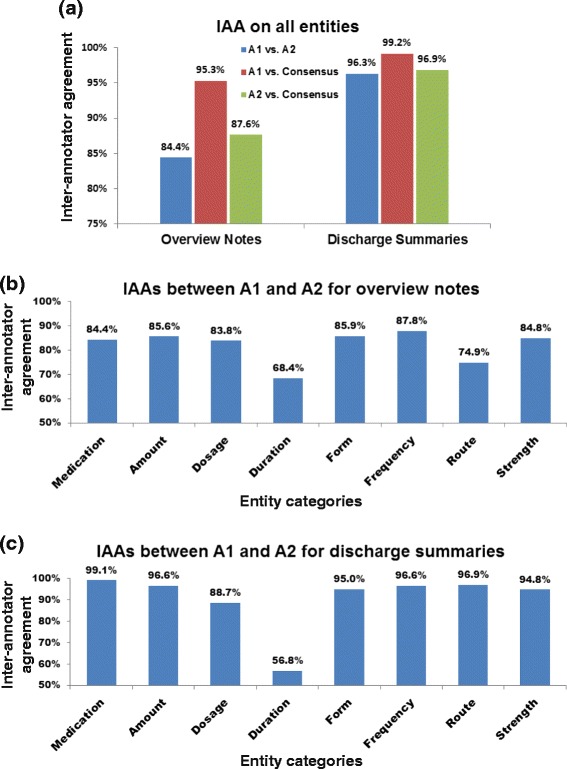
The overall inter-annotator agreements (IAAs; F-value) for overview notes and discharge summaries **(a)**. The IAAs on individual entity categories are also presented **(b and c)**.

Figure 4 shows the IAAs between the annotators and between the annotators and the consensus. The IAAs were computed for overall (Figure 4a) and for each medication entity category per note type (Figure 4b and c). We observed that the IAAs were generally lower on the overview notes due to irregular medication expressions made by physicians. For instance, in certain cases the medication “Augmentin” was written as “abx” (antibiotics) in the clinical notes but “amoxicillin clavulanate” in the prescription list, making it difficult for annotator 2 (with less clinical experience) to identify the match. The IAAs on duration were also low due to the frequency of this category (Table 1). Nevertheless, the consensus made under the supervision of the annotation manager still assured the high quality of the gold-standard set, which was evident by the high IAAs between the annotator 1 (clinical research nurse) and the consensus (Figure 4a).

### Medication entity detection and attribute linkage

Table 2 shows the performances of the medication entity detection process. The ML-based algorithm achieved an overall F-value of 93.3% (95.0% precision, 91.6% recall). Most of the entity categories achieved greater than 90% F-values. The performance was low on “duration” because of the limited amount of training data in this category (Table 1).

**Table 2:** **Performance of the entity detection process**

**Process**	**Medication entity detection**		
**Category**	**P [%]**	**R [%]**	**F [%]**
Medication name	92.3	88.6	90.4
Amount	94.8	90.7	92.7
Dosage	90.9	87.9	89.4
Duration	73.5	43.2	54.5
Form	95.0	93.0	94.0
Frequency	94.0	89.5	91.7
Route	95.5	94.0	94.7
Strength	95.0	94.9	95.0
Overall	95.0	91.6	93.3

P indicates precision; R recall; F F-value.

The rule-based attribute linkage achieved the overall F-value of 99.1% (precision 98.7%, recall 99.4%, Table 3). When combining the entity detection and the attribute linkage processes (processes 1 and 2 in Figure 2), the propagated F-value was 91.2% (precision 92.8%, recall 89.6%) across all attributes.

**Table 3:** **Performance of the attribute linkage process**

**Process**	**Attribute linkage**			**Ent. Det. + Att. Link.**		
**Category**	**P [%]**	**R [%]**	**F [%]**	**P [%]**	**R [%]**	**F [%]**
Amount	97.5	99.3	98.4	92.8	89.5	91.1
Dosage	97.8	99.5	98.7	87.9	86.1	87.0
Duration	94.3	96.6	95.4	73.4	42.1	53.5
Form	99.2	99.6	99.4	94.2	91.9	93.0
Frequency	99.0	99.2	99.1	92.4	87.6	89.9
Route	99.3	99.3	99.3	94.5	92.1	93.3
Strength	99.0	99.8	99.4	93.9	93.3	93.6
Overall	98.7	99.4	99.1	92.8	89.6	91.2

P indicates precision; R recall; F F-value.

### Medication matching

Table 4 shows the performances of the hybrid medication matching on gold-standard matched and discrepant medications respectively. The medication name matching achieved 97.5% F-value on matched medications and 85.5% on discrepant medications. The attribute matching achieved similar performances on matched categories, while the performances were reduced on discrepant categories. The overall F-value on the matched categories was 95.3% (precision 98.1%, recall 92.8%), higher than 64.7% F-value on the discrepant categories (precision 53.4%, recall 81.8%).

**Table 4:** **Performance of the medication matching process**

**Process**	**Medication matching**						**Ent. Det. + Att. Link. + Med. Mat.**					
	**Matched**			**Discrepant**			**Matched**			**Discrepant**		
**Category**	**P [%]**	**R [%]**	**F [%]**	**P [%]**	**R [%]**	**F [%]**	**P [%]**	**R [%]**	**F [%]**	**P [%]**	**R [%]**	**F [%]**
Medication name	96.8	98.2	97.5	88.6	82.5	85.5	92.4	90.7	91.5	71.5	65.2	68.2
Amount	98.8	91.7	95.1	39.8	82.6	53.7	98.4	86.3	92.0	34.2	68.7	45.7
Dosage	97.8	90.2	93.9	41.2	77.4	53.8	97.2	83.0	89.6	29.1	54.3	37.9
Duration	98.6	82.9	90.1	17.6	75.0	28.6	100	40.5	57.6	2.2	9.1	3.5
Form	99.4	90.8	95.0	8.2	61.7	14.4	99.3	86.6	92.5	5.3	39.2	9.4
Frequency	98.3	83.0	90.0	30.0	83.0	44.1	98.0	77.0	86.2	25.1	68.6	36.8
Route	99.7	90.1	94.7	19.7	89.5	32.3	99.4	86.3	92.4	16.9	72.8	27.4
Strength	99.5	93.9	96.6	40.0	89.5	55.3	99.2	89.3	94.0	30.1	76.6	43.2
Overall	98.1	92.8	95.3	53.4	81.8	64.7	95.9	86.6	91.0	42.2	64.6	51.0

P indicates precision; R recall; F F-value.

By integrating all three processes, the proposed medication discrepancy detection algorithm (Figure 2) achieved 92.4%/90.7%/91.5% (precision/recall/F-value) on matching names of matched medications and 71.5%/65.2%/68.2% (precision/recall/F-value) on discrepant medications. The overall performance was 95.6%/86.6%/91.0% (precision/recall/F-value) on the matched categories and 42.2%/64.6%/51.0% (precision/recall/F-value) on the discrepant categories.

### Recall propagation on discrepant medications

**Figure 5:**
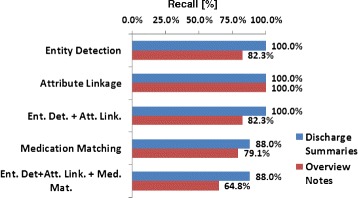
The recalls of medication name detection on discrepant medications.

To assess the influence of each algorithm component on discrepant medication detection, we plotted in Figure 5 the recall propagation during the three processes. The recalls were calculated on discrepant medications from the overview notes and the discharge summaries respectively. For discrepant medications in the discharge summaries, the proposed algorithm achieved a 100% recall on entity detection and an overall recall of 88.0% after medication matching. The performance was lower on the overview notes, with 82.3% on entity detection and 64.8% after medication matching. Since the attribute linkage process only linked the attributes to the identified medication names, it did not influence the recall of medication name detection (i.e. recall = 100%).

## Discussion

### Algorithm performance

We evaluated the three processes of automated medication discrepancy detection individually and in combination using double-annotated, gold-standard medication reconciliation data. The ML-based medication entity detection showed good capability in identifying medication names and attributes and achieved an F-value of 93.3% (Table 2). The results of attribute linkage suggested that even a relatively rudimentary, rule-based algorithm could yield high performance (overall F-value 99.1%, Table 3). The hybrid medication matching also achieved reasonable recalls on medication name matching (98.2% on matched medications and 82.5% on discrepant medications, Table 4), suggesting the effectiveness of the NLP and information extraction techniques used such as the RxNorm dictionary mapping. The attribute matching achieved similar performances on the matched categories. The performances were reduced on matching discrepant attributes, resulting in a lower overall performance on the discrepant categories.

By integrating the three processes, the proposed algorithm achieved a much improved recall (90.7%/65.2% on matched/discrepant medications, Table 4) over the recalls reported in the literature (e.g. 23.4% on matched medications reported in the work of Schnipper et al. [19]), while keeping the precision at a manageable level (92.4%/71.5% on matched/discrepant medications). The recall propagation (Figure 5) showed that both the entity detection and medication matching processes contributed to the loss of detection on discrepant medications; therefore, further refinements in these components are required to improve medication discrepancy detection. In addition, the algorithm achieved better recalls on detecting discrepant medications in the discharge summaries. This is because the institutional EHR system contains a smart list function to facilitate medication entry on discharge summaries, where the physicians could directly pull structured medications from a patient’s medication list, resulting in more controlled vocabularies in the discharge summaries. The observation suggests that encouraging physicians to utilize structured medication inputs in the clinical workflow could benefit computerized medication reconciliation.

### Error analysis, limitations and future work

Since the study focused on detecting discrepant medications, we performed error analysis by reviewing all false negatives made by the algorithm (i.e. discrepant medications identified by the annotators but missed by the algorithm). The hybrid algorithm made 397 errors, which were grouped into six categories in Table 5. Approximately 68% of the errors (cause 1) were ascribed to the omission of medication entities by the entity detection algorithm due to abbreviations used in the clinical notes (e.g. “abx” for “antibiotics” and “NS” for “normal saline”), misspellings (e.g. misspelled “Affrin” as “Afrin” and “Nutropin” as “Neutropen”) and uncommon medication names (e.g. “Pedia Sure”). This observation suggests the limitation of the data-driven ML-based algorithms. In our future work, we will investigate using NLP-based pre-processing for abbreviation extension and spelling correction to see if they improve the accuracy in medication detection.

**Table 5:** **False negative errors made by the medication discrepancy detection algorithm**

**Cause of false negative errors identified by the chart review**	**Error [%]**
1. The medication was omitted by the medication entity detection algorithm	68.0%
2. The medication was matched to a wrong medication due to similar medication names (e.g. methylprednisolone and prednisolone)	9.1%
3. The prescription contains more ingredients than the medication in the clinical note or vice versa (e.g. albuterol vs. ipratropium albuterol)	6.3%
4. The medication in the clinical note was matched to a correct prescription (e.g. matching diastat to diazepam) but the prescription had a different route (e.g. oral route vs. rectal route)	6.0%
5. The medication and the prescription names co-occurred in the same RxNorm description as ingredients rather than synonyms (e.g. “glycerin” and “polyethylene glycol” co-occurred in the RxNorm description of “artificial tears”)	4.8%
6. Other reasons	5.8%

Another set of errors (cause 2 and 3) was caused by the confusion between similar medication names (e.g. match the medication “albuterol” to “ipratropium albuterol”). This is because the current medication matching algorithm uses “bag-of-words” patterns, which limits its ability in accurately matching combination medications. To alleviate this problem, we will extend the pattern set to “bag-of-phrases” in the algorithm in our future study. In addition, we observed that the annotators tended to determine a medication-prescription pair as discrepant even if the medication was the same but with a different route (e.g. oral route vs. rectal route). This caused another 6% of false negatives (cause 4) because the matching algorithm processed medication names and their attributes separately. In the future we will add this knowledge-based rule in the algorithm. Additional rules are also required to tune the RxNorm dictionary mapping to reduce inappropriate matches that would cause unexpected errors (cause 5).

There are limitations of our study. One limitation is that the performance of attribute matching is still low because some attributes were embedded in the context that could be difficult to understand by the current algorithm. For instance, “Baclofen 5 mg tab po bid”, “Baclofen 5 mg tab 1 tab po at 6 AM, 1 tab at 2 PM” and “Baclofen 10 mg tab” in the clinical notes suggested the same amount (10 mg) of Baclofen for treatment. However, since the algorithm could not analyze the semantics in the context, it failed to match the first two cases with the correct amount in the prescription list. The same failures were also observed on frequency and dosage, such as identifying “AM” and “PM” in “taking sucralfate 2 ml AM and 2 ml PM” as frequency but failing to match it to “twice a day” in the prescription list. In our future work we will apply advanced NLP and knowledge-based algorithms to account for the rich semantic context and high variance to improve the accuracy of attribute matching.

Another limitation of the study is that its evaluation is restricted to retrospective data. The prototype needs to be transferred to a production environment to adequately estimate the practicality of automated medication discrepancy detection. Finally, the patient population investigated in the study usually has long medication lists documented in clinical notes, providing a potentially more suitable foundation for medication entity and discrepancy detection algorithms. To study its generalizability, we plan to test the algorithm on a more diversified patient population (e.g., patients in general care settings), multiple institutions, and clinical data under different formats (e.g. clinical record formats used in different vendors’ EHR products).

## Conclusion

By leveraging ML and NLP technologies, we developed and implemented an end-to-end hybrid algorithm for reconciling medications between free-text clinical notes (unstructured data) and discharge prescription lists (structured data). In a double-annotated, gold-standard based evaluation of real-world medication reconciliation data, the proposed algorithm showed good capability in medication entity detection, attribute linkage and medication matching. The algorithm achieved 92.4%/90.7%/91.5% (precision/recall/F-value) on identifying matched medications in the gold-standard and 71.5%/65.2%/68.2% (precision/recall/F-value) on discrepant medications. Further refinements in the algorithm are required to increase the recall in identifying discrepant medications. However, even at this early stage of development, automated medication discrepancy detection shows a promising outcome in assisting medication reconciliation. Consequently, we hypothesize that the computerized algorithm, when transferred to the production environment, will have potential for significant impact in reduction of effort for conducting medication reconciliation in the clinical practice setting.

## Additional file

Additional file 1: **Table A.1 Pseudo-code of the attribute linkage algorithm (Process 2).**

## Abbreviations

CCHMC: Cincinnati Children’s Hospital Medical Center
CCMHP: Cincinnati Children’s Medical Home Program
CRF: Conditional random field
EHR: Electronic Health Record
IAA: Inter-annotator agreement
NLP: Natural language processing
ML: Machine learning
P: Precision
R: Recall
F: F-value
