# Supplementary material for: An end-to-end hybrid algorithm for automated medication discrepancy detection
Source: BMC Med Inform Decis Mak. 2015 May 6;15:37. doi: 10.1186/s12911-015-0160-8 (PMC4427951; doi:10.1186/s12911-015-0160-8)
Supplement: Supplementary file 1 — Table A.1 Pseudo-code of the attribute linkage algorithm (Process 2). [file 12911_2015_160_MOESM1_ESM.docx]

**Additional file**

**Table A.1** Pseudo-code of the attribute linkage algorithm (Process 2).

| **Input**: medication entities ${\{e_{i}\}}_{i=1}^{N}$ identified by entity detection and the corresponding start/end positions in the text ${\{s_{i},t_{i}\}}_{i=1}^{N}$  1) **Initialization**  sort the entities by the start positions  set the linkage information set $attribute\_linkage=\{\}$  2) **For** $i=1 to N$  **If** $e_{i}$ is a medication attribute **then**  **For** $j=i to 1$  **If** $e_{j}$ is a medication name **then**  $previous\_medication=j$  $distance\_to\_p=s_{i}-t_{j}$  Break  **End if**  **End for**  **For** $j=i+1 to N$  **If** $e_{j}$ is a medication name **then**  $next\_medication=j$  $distance\_to\_n=s_{j}-t_{i}$  Break  **End if**  **End for**  **If** $distance\_to\_p \leq distance\_to\_n$ **then**  $attribute\_linkage\{i\}=previous\_medication$  **Else**  $attribute\_linkage\{i\}=next\_medication$  **End if**  **End if**  **End for**  **Return**: the linkage information set $attribute\_linkage$ |
| --- |
